# Exosome‐transmitted circ_MMP2 promotes hepatocellular carcinoma metastasis by upregulating MMP2

**DOI:** 10.1002/1878-0261.12637

**Published:** 2020-05-06

**Authors:** Dengrui Liu, Hongxia Kang, Mingtai Gao, Li Jin, Fang Zhang, Dongqin Chen, Mianli Li, Linghui Xiao

**Affiliations:** ^1^ Department of Pediatric Surgery the First Hospital of Lanzhou University China; ^2^ Department of Anesthesiology Gansu Provincial Hospital Lanzhou China; ^3^ Department of Radiotherapy Sichuan Cancer Hospital & Institute Sichuan Cancer Center School of Medicine University of Electronic Science and Technology of China Chengdu China; ^4^ Laboratory for Advanced Interdisciplinary Research the First Affiliated Hospital of Wenzhou Medical University China; ^5^ Department of Medical Oncology Jiangsu Cancer Hospital & The Affiliated Cancer Hospital of Nanjing Medical University China; ^6^ Oncology Department Binzhou Medical University Hospital China; ^7^ Infectious Disease Department Hospital of Chengdu University of Traditional Chinese Medicine China

**Keywords:** circ_MMP2, exosome, HCC, metastasis, MMP2

## Abstract

Exosomes released by tumor cells have been recently identified as important determinants of tumor progression. They often carry circular RNAs that are differentially expressed in tumors and may regulate tumorigenesis and metastasis. Here, we showed that supernatant of 97H hepatocellular carcinoma (HCC) cell line could promote metastasis in L02 human liver cells and HCC cell lines. Moreover, we determined that circ_MMP2 (has_circ_0039411) could be delivered by 97H‐ or LM3 cell‐derived exosomes to L02 and HepG2 cell cultures. High expression of circ_MMP2 led to the upregulation of its host gene matrix metallopeptidase 2 (MMP2) via the sponging of miR‐136‐5p. Rescue assays demonstrated that miR‐136‐5p and MMP2 were two essential participants in HCC metastasis. Finally, high level of circ_MMP2 or MMP2, as well as low level of miR‐136‐5p, was correlated with low overall survival of HCC patients. Our study highlights a novel molecular pathway in HCC cell‐derived exosomes.

## Graphical abstract

97H and LM3 cell line cocultured together had strong metastasis capacity. They released exosomes to produce circ_MMP2 to sponge miR‐136‐5p by enhancing MMP2 to accelerate the process hepatocellular cancinoma.

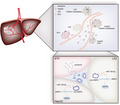

**Abbreviations**
ceRNA: competing endogenous RNA
circRNAs: circular RNAs
CM: culture medium
EMT: epithelial–mesenchymal transition
HCC: hepatocellular carcinoma
MMP2: matrix metallopeptidase 2
NTA: Nanoparticle Tracking Analysis
qRT–PCR: quantitative real‐time PCR
RIP: RNA immunoprecipitation

## 1 Introduction

Hepatocellular carcinoma (HCC) is acknowledged as the third common malignant tumor worldwide (Singal and El‐Serag, 2015). Liver fibrosis and cirrhosis are two major reasons for the initiation of HCC (Bruix *et al.*, 2014). Recently, surgery resection and liver transplantation are still the main curative methods for the treatment of HCC, which limits the overall survival of HCC patients (European Association for the Study of the Liver, European Organisation for Research and Treatment of Cancer, 2012; Yu *et al.*, 2019). Hence, exploring novel molecular mechanism associated with the malignant development of HCC is very essential for the detection of novel therapeutic strategy.

Exosomes are the important media for cell–cell communication. Exosomes are small, nanometer‐sized (50–100 nm) vesicles originated from endocytosis, which can be released by cells and transferred into extracellular milieu under physiological or pathological condition. Recently, exosomes have been reported to regulate tumor progression by carrying or delivering a variety of biological modulators, including miRNAs, mRNAs, and proteins (Falcone *et al.*, 2015; Paladini *et al.*, 2016; Regev‐Rudzki *et al.*, 2013; van der Pol *et al.*, 2012). Exosomes derived from tumor cells are crucial participants in biological processes, such as epithelial–mesenchymal transition (EMT) and tumor metastasis (Azmi *et al.*, 2013; Junttila and de Sauvage, 2013; Zhou *et al.*, 2018). Increasing evidence revealed that thousands of mammalian genome encodes circular RNAs (circRNAs) (Sanger *et al.*, 1976; Westholm *et al.*, 2014). circRNAs are characterized by covalently closed loops and without protein‐coding capacity (Memczak *et al.*, 2013). Recent literatures have elucidated that circRNAs are biological participants in diverse physiological and pathological processes (Li *et al.*, 2018b; Xu *et al.*, 2018). Especially, the roles of circRNAs in tumor progression are widely investigated in recent years (Hsiao *et al.*, 2017a; Song *et al.*, 2016; Yang *et al.*, 2017). Mechanistically, circRNAs can function as competing endogenous RNAs (ceRNAs) to upregulate mRNAs via sequestering miRNAs in human cancers (Xu *et al.*, 2017; Zhong *et al.*, 2017, 2018). Thus, investigating the roles of circRNAs in HCC progression is necessary to discover novel therapeutic strategy.

The aim of this study was to classify the mechanism underlying the exosome‐delivered circ_MMP2 released by HCC cell lines. The malignant degree of normal cell line and HCC cell lines was analyzed. Moreover, the culture medium for 97H cell line (97H‐CM) was used to treat normal cell line and HCC cell line. The metastatic ability was found to be stronger after cells were treated with 97H‐CM. Further investigation was used to identify the exosomes derived from 97H or LM3 cell line transferred circ_MMP2. circ_MMP2 was identified to be located in cytoplasm and potentially exerted ceRNA functions. The function of ceRNA pathway was identified in this study by rescue assays. Our research findings may contribute to offer new potential therapeutic strategies for HCC.

## 2 Materials and methods

### 2.1 Clinical specimens

Metastatic or nonmetastatic tissues were collected from HCC patients after resection in the First Hospital of Lanzhou University. All procedures of this study were approved by the Ethical Committee of the First Hospital of Lanzhou University. Before this study, every patient enrolled in this study had given the written informed consent.

### 2.2 Cell culture and transfections

Four human liver cancer cell lines (97H, LM3, SMMC‐7721, and HepG2) and human normal liver cell line L02 were purchased from Cell Bank of Type Culture Collection of the Chinese Academy of Sciences (Shanghai Institute of Cell Biology). DMEM (Gibco, Grand Island, NYC, USA) supplemented with 10% FBS (Gibco) was used as the culture medium for all HCC cell lines. RPMI‐1640 medium (Gibco) supplemented with 10% FBS (Gibco) was used for L02 cell culture. Cell lines were cultured in a humidified incubator with 5% CO_2_ at 37 °C. Short tandem repeat profiling was used to authenticate cell lines were free from mycoplasma.

circ_MMP2 was stably silenced by using specific shRNAs targeting circ_MMP2 (sh/circ_MMP2#1, sh/circ_MMP2#2, or sh/circ_MMP2#3). Nonspecific shRNA was used as negative control (shCtrl). shRNAs were synthesized by GenePharma (Shanghai, China). miR‐136‐5p mimics, inhibitor, or pcDNA3.1 vector containing the whole sequence of MMP2 or empty pcDNA3.1 vector was obtained from RiboBio Company (Guangzhou, China). All plasmid sequences are listed in Table S1. Lipofectamine 2000 (Invitrogen, Carlsbad, CA, USA) was used for all transfections.

### 2.3 Quantitative real‐time PCR analysis

Total RNA was isolated with TRIzol reagent (Invitrogen), either from frozen tissues or from cultured cells. Reverse transcription of total RNA (1 μg) was made by using the Superscript III transcriptase (Invitrogen). Bio‐Rad CFX96 system (Bio‐Rad, Hercules, CA, USA) with SYBR green was used to conduct RT–qPCR, thereby examining the expression level of genes. RNA expression was normalized to GAPDH. Primers for circ_MMP2 were obtained in accordance with the head‐to‐tail junction. PCR primers are provided in Table S1.

### 2.4 Western blotting analysis

Whole proteins extracted from cell lines were lysed in RIPA buffer and were centrifuged at 4000 ***g*** for about 15 min. Bicinchoninic acid assay was performed to determine protein concentrations. Proteins separated on 12% SDS/PAGE gel were removed onto PVDF membranes (Millipore, Billerica, MA, USA). After blocking, specific primary antibodies against E‐cadherin (ab133597, 1 : 1000), N‐cadherin (ab245117, 1 : 1000), MMP2 (ab215986, 1 : 1000), and GAPDH (ab181602, 1 : 2000) were used to incubate membranes. All antibodies used in this study were purchased from Abcam (Cambridge, UK). Then, the membranes were incubated with HRP‐conjugated secondary antibodies and visualized using ECL system (Thermo Fisher Scientific, Rochester, NY, USA).

### 2.5 Transwell invasion assay and wound‐healing assay

Invasive cells were measured by using transwell Matrigel assay. At first, 5 × 10^4^ L02 and HepG2 cells were plated into 24‐well plates with required inserts (Corning, Corning, NY, USA). The inserts contained FBS‐free medium. However, medium supplemented with 10% FBS was put into the outside of the inserts. Then, the inserts were added with equal amounts of 97H‐ or LM3 cell‐derived exosomes. Twenty‐four hours later, the inserts were fixed and stained with crystal violet in accordance with the user guidance. The number of invasive cells was calculated after representative fields were photographed.

Migratory ability of cells was measured by wound‐healing assay. Firstly, L02 or HepG2 cells at equal numbers were plated into six‐well plates. Next, the cell monolayers on the plates were wounded by using a pipette tip to make a gash. Twenty‐four hours later, wound healing was measured under a microscope by observing the cells that migrated into the clear gash.

### 2.6 Animal studies

To examine the tumor formation ability of different cell lines, 1 × 10^6^ L02 or HCC cell lines were injected into the body of male nude mice via subcutaneous injection. Tumor growth was measured every 4 days with digital calipers. Twenty‐eight days later, the mice were sacrificed and tumors were resected. Tumor growth was monitored every 4 days. Tumor weight and tumor volume were measured. Animal experiments conducted in this study had been approved by the First Hospital of Lanzhou University. Tissues isolated from the nude mice in different groups were subjected to immunohistochemistry.

### 2.7 *In vivo* metastasis assay

Metastatic nodules were calculated as previously described (Xiao *et al.*, 2019). Metastatic lung was detected with hematoxylin and eosin staining.

### 2.8 Isolation and analysis of exosomes

Isolation of exosomes was performed in accordance with a previous study (Kornilov *et al.*, 2018). Exosome was isolated from equal numbers of different cells that were cultured in 10‐cm plates containing fresh DMEM supplemented with serum. Exosome was depleted by centrifugation at 120 000 ***g*** for 1 h. Forty‐eight hours later, 0.22‐μm filters (Millipore) were used to filtrate CM for collection. Exosomes in CM were isolated by ultracentrifugation experiments with Optima MAX‐XP (Beckman Coulter, Pasadena, CA, USA). Exosomes were observed under a Philips CM120 BioTwin transmission electron microscope (FEI Company, Hillsboro, OR, USA).

### 2.9 PKH67 staining

As previously described (Ren *et al.*, 2019), exosomes (5 μg) were probed by using PKH67 labeling KH67 Fluorescent Cell Linker Kits (Sigma‐Aldrich, St. Louis, MO, USA). At first, labeled exosomes uptake into recipient cells were collected from 100 mL of culture medium. DMEM with or without PKH67‐labeled exosome solution was added to each well so as to assess the uptake of exosomes into recipient cells.

### 2.10 Transmission electron microscopy

For this assay, first 0.2 m phosphate buffer (200 μL) was used for exosome pellet resuspension followed by mixture of 4% paraformaldehyde with an equal volume (1 : 1). Then, we pipette 5 μL aliquot from each specimen onto UV‐treated nickel electron microscopy grids (200 mesh) coated with formvar/carbon and let it absorb for about 20 min. 2.5% glutaraldehyde was utilized for fixating exosomes for 5 min, followed by 2% uranyl acetate treatment for 10 min. Eventually, Hitachi H‐7600 transmission electron microscope analysis was conducted at 80 kV.

### 2.11 Nanosight particle tracking

Collected exosome samples were diluted utilizing sterile PBS at 1 : 50, and then, they got injected into Nanosight NS300 unit (Malvern Instruments, Westborough, MA, USA). Following the guide's recommendations, we manually operated capture and analysis settings. Laser light scattering was utilized for particle visualization, followed by a Brownian motion capture through a digital video. Following, the videotapes were recorded, nanoparticle tracking analysis (nta) 2.3 software (Shanghai XP Biomed Ltd., Shanghai, China) was used for later analysis tracking more than 200 individual particles each run. On the basis of light scattering and Brownian motion, the software produced size distribution profiles with high resolution and measured the concentration for the tracked particles.

### 2.12 Immunofluorescence

Antibodies against CD63, CD81, and secondary antibodies with Alexa Fluor^®^ 594 (1 : 1000; Life Technologies, Thermo Fisher Scientific, Waltham, MA, USA) were used for immunofluorescence staining. DAPI was used to stain the nucleus as a control. Images were visualized by using the FV‐1200 laser scanning confocal microscope.

### 2.13 Luciferase analysis

Cells plated in 24‐well plates were transfected with Lipofectamine 3000 (Invitrogen). pRL‐TK was taken as an internal control. Based on the manufacturer's manual, Dual‐Luciferase Assay (Promega, Madison, WI, USA) was used for the detection of luciferase activity. To ascertain the influence of circ_MMP2 on the transcriptional activity of MMP2, pGL3 luciferase reporter assay was conducted. We introduced MMP2 promoter into the pGL3 plasmid vector (Promega). L02, HepG2, and 293T were cotransfected with the luciferase constructs described above and the circ_MMP2‐overexpressing vector or normal control (NC). A Luciferase Reporter Assay Kit (Promega) was used to reflect the MMP2 promoter activity.

### 2.14 RNA immunoprecipitation

According to the previous study (Cai *et al.*, 2019), Ago2‐RIP assay was conducted to validate the interaction between circ_MMP2 and miR‐136‐5p as well as between MMP2 and miR‐136‐5p.

### 2.15 RNA pull‐down assay

pAd‐Track‐cmv‐circ_MMP2 was transfected into 97H‐CM‐cultured HepG2 cells to overexpress circRNA_MMP2. Total RNA was isolated from 97H‐CM HepG2 cell transfected with circ_MMP2. About 100 μg total RNA was used to incubate streptavidin magnetic beads (500 μg, S1421S; NEB, Ipswich, MA, USA) which had been incubated with 200 pmol biotin‐miR‐874‐3p, biotin‐miR‐136‐5p, or biotin‐miR‐299‐3p. Finally, the binding RNA was eluted and subjected to RT–qPCR assay for circ_MMP2 detection.

### 2.16 Statistical analysis

Data were analyzed using the spss software version 16.0 (IBM, Armonk, NY, USA). Results obtained from three independent experiments were presented as mean ± SD. Statistical significance was evaluated by using Student's *t*‐test and one‐way ANOVA. Expression correlation between genes was analyzed with Pearson's correlation analysis. Statistical significance of data was identified when *P* value less than 0.05.

## 3 Results

### 3.1 Metastatic ability of human normal liver cell line and HCC cell lines

**Figure 1:**
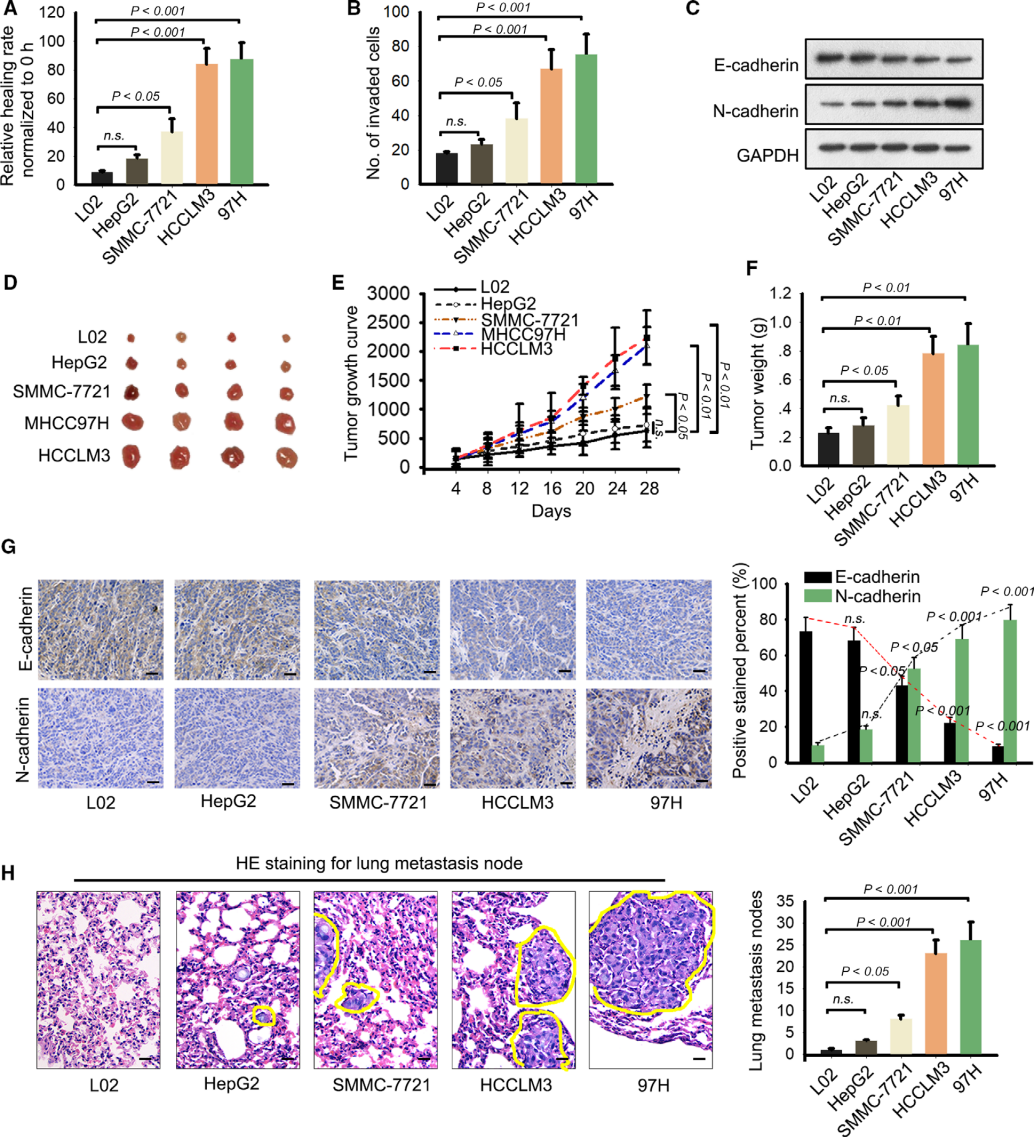
Metastatic ability of human normal liver cell line and HCC cell lines. (A, B) The migratory and invasive ability of one human normal liver cell line and four HCC cell lines was assessed by wound‐healing and transwell invasion assays (mean ± SD; *n* = 6; one‐way ANOVA). (C) Western blotting was used to identify EMT‐related proteins (E‐cadherin and N‐cadherin) in different cell lines. (D) Tumors derived from normal liver cell line or four HCC cell lines were resected. (E, F) Tumor volume (mean ± SD; *n* = 4; two‐way ANOVA) and tumor weight (mean ± SD; *n* = 4; one‐way ANOVA) in different groups were calculated and shown. (G) IHC staining indicated that HCC cell lines presented the lower positivity of E‐cadherin and higher positivity of N‐cadherin than normal cell line (scale bar = 50 μm; mean ± SD; *n* = 4; one‐way ANOVA). (H) *In vivo* metastatic detection was applied to test lung metastasis nodes in HCC group and normal group (scale bar = 50 μm; mean ± SD; *n* = 4; one‐way ANOVA). *P* < 0.05, *P* < 0.01, and *P* < 0.001 indicated data were statistically significant. n.s., no significance.

At first, the migratory and invasive ability of one human normal liver cell line and four HCC cell lines was assessed by wound‐healing and transwell invasion assays. According to the experimental result, migratory ability of HCC cell lines was stronger than that of normal cell line (Fig. 1A,B). EMT is an important biological process that is closely associated with cell migration and invasion. Here, western blotting was used to identify EMT‐related proteins (E‐cadherin and N‐cadherin) in different cell lines. Unsurprisingly, decreased E‐cadherin level and increased N‐cadherin level were detected in HCC cell lines compared with the normal cell line (Fig. 1C). Furthermore, all these five cell lines were injected into nude mice. Twenty‐eight days later, we observed tumor growth in different groups. The results showed that tumors derived from HCC cell lines were bigger than those derived from normal cell lines (Fig. 1D). Tumor volume and tumor weight presented the same tendency with tumor size (Fig. 1E,F). Subsequently, IHC staining indicated that HCC cell lines presented the lower positivity of E‐cadherin and higher positivity of N‐cadherin than normal cell line (Fig. 1G). *In vivo* metastatic detection showed lung metastasis nodes in HCC group more than that in normal group (Fig. 1H). Taken together, we identified that the metastatic ability of HCC cell lines, especially 97H and LM3 cell lines, is significantly stronger than normal liver cell line.

### 3.2 High metastasis was observed in cell lines treated with 97H‐CM

**Figure 2:**
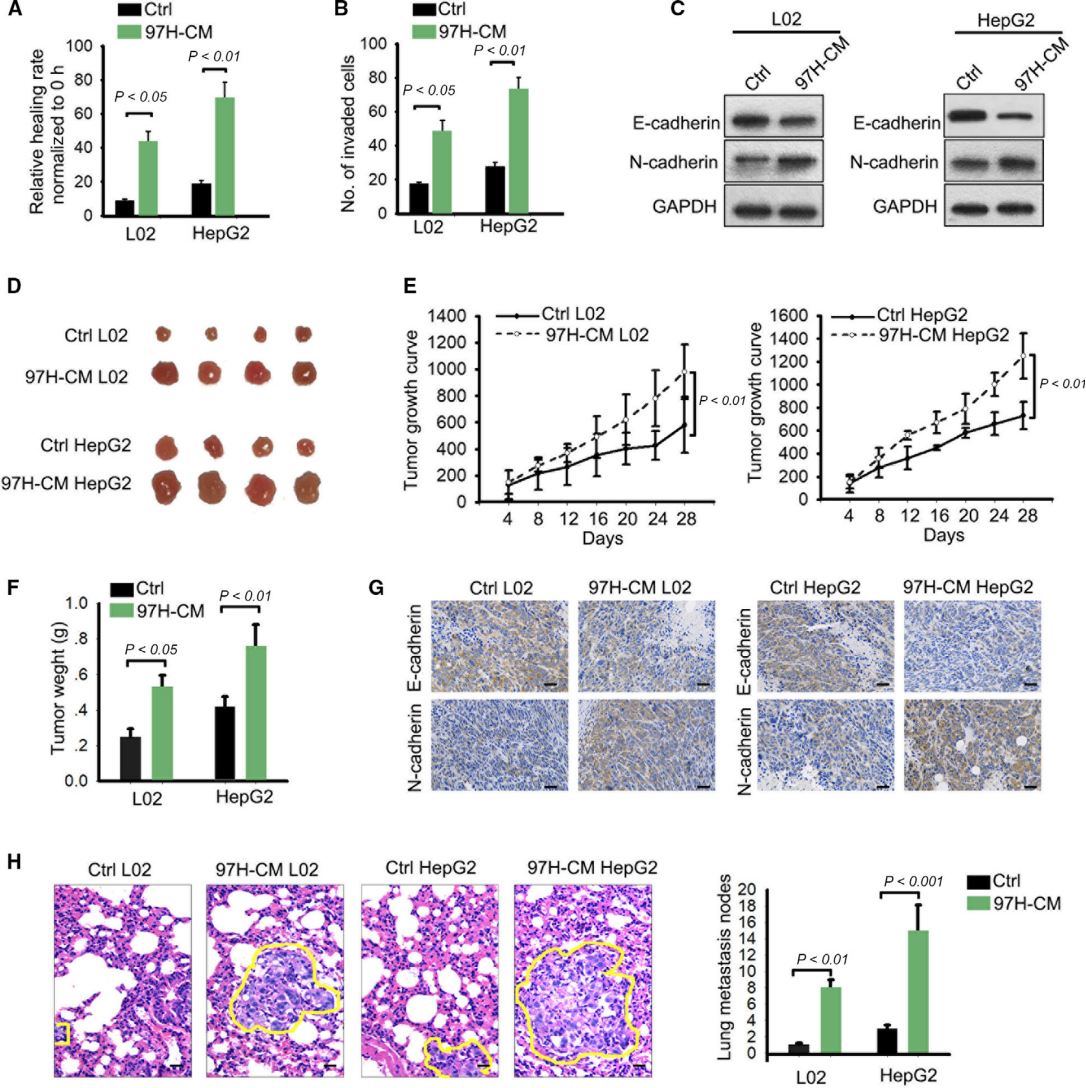
High metastasis was observed in cell lines treated with 97H‐CM. (A, B) Migration and invasion in L02 and HepG2 cell lines treated with or without 97H‐CM (mean ± SD; *n* = 6; one‐way ANOVA). (C) The levels of E‐cadherin and N‐cadherin in 97H‐CM group or original group. (D) Animal study revealed that tumor growth in 97H‐CM group was faster than control group. (E, F) Tumor volume (mean ± SD; *n* = 4; two‐way ANOVA) and tumor weight (mean ± SD; *n* = 4; Student's *t*‐test) in different groups were measured. (G) Positivity of E‐cadherin or N‐cadherin in 97H‐CM group or control group was assessed by IHC staining (scale bar = 50 μm; *n* = 4). (H) Lung metastasis nodes for 97H‐CM group or control group were measured and counted (scale bar = 50 μm; mean ± SD; *n* = 4; Student's *t*‐test). *P* < 0.05, *P* < 0.01, and *P* < 0.001 indicated data were statistically significant.

Considering the highest metastasis of 97H, the L02 cell line and HepG2 cell line were treated with the culture medium of 97H (97H‐CM). Similarly, we applied *in vitro* and *in vivo* experiments to test metastatic ability. Interestingly, we found that the migratory and invasive ability of original L02 and HepG2 cell lines was weaker than cell lines treated with 97H‐CM (Fig. 2A,B). Additionally, we determined that there were relative lower level of E‐cadherin and higher level of N‐cadherin in 97H‐CM group compared with the original cell lines (Fig. 2C). Animal study revealed that tumor growth in 97H‐CM group was faster than that in control group (Fig. 2D). Consistently, tumor volume and tumor weight presented the same tendency (Fig. 2E,F). And 97H‐CM group exhibited lower E‐cadherin positivity and higher N‐cadherin positivity compared with control group (Fig. 2G). Finally, we observed the lung metastatic nodes in different groups. As expected, lung metastasis nodes of 97H‐CM group were observed more than that in control group (Fig. 2H). Therefore, we confirmed that 97H‐CM‐cultured cell lines had strong metastatic ability.

### 3.3 circ_MMP2 was transferred by LM3 and 97H‐derived exosomes

**Figure 3:**
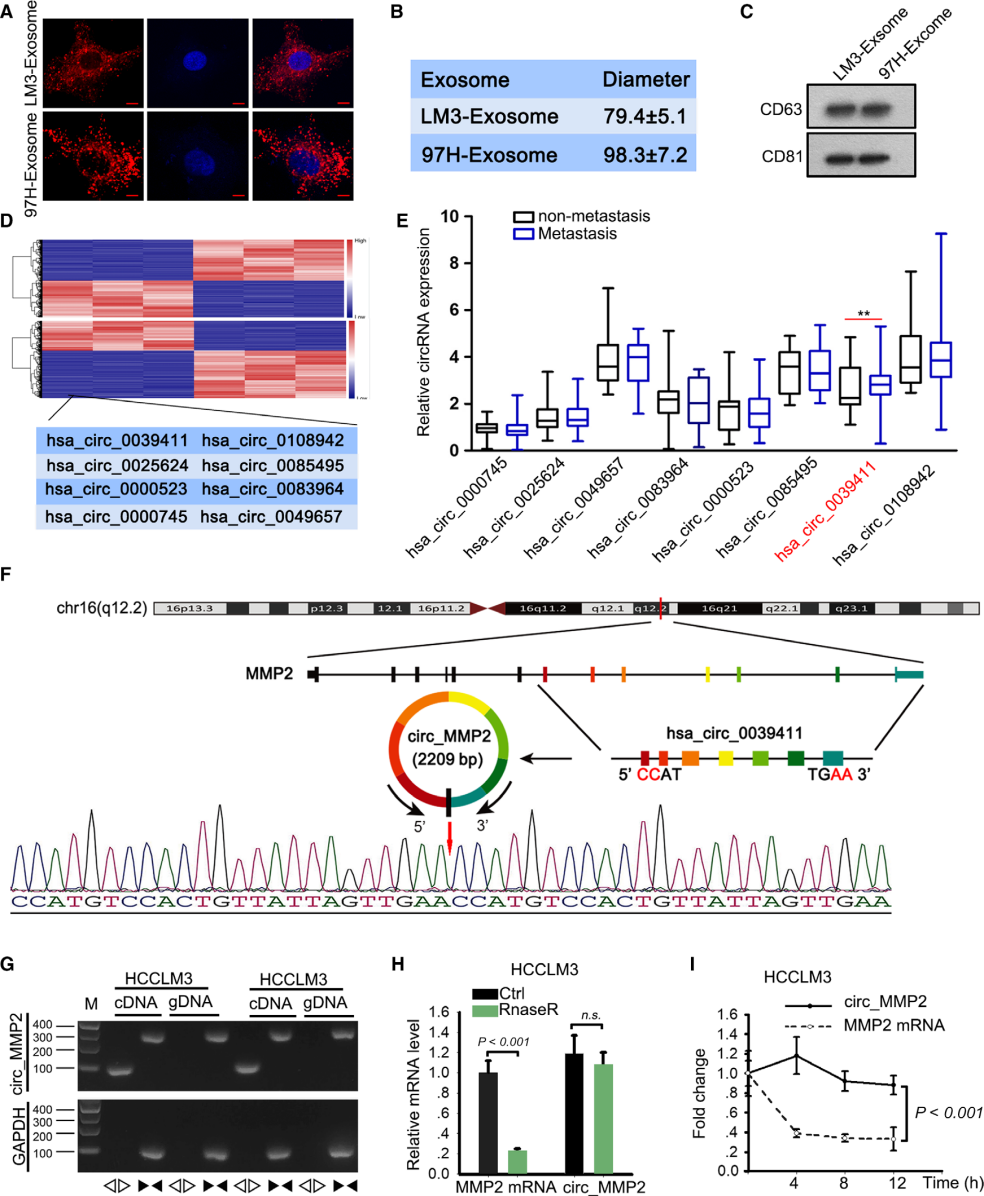
circ_MMP2 was transferred by LM3 and 97H‐derived exosomes. (A, B) The cup‐shaped structure and size of exosomes released by 97H and LM3 cells were assessed by PKH67 staining (scale bar = 10 μm; *n* = 5) and NTA. (C) The isolated particles were verified to be exosomes via detection of characteristic CD63 and CD81. (D) 38 miRNAs that can bind with both circ_MMP2 and MMP2 were subjected to qRT–PCR analysis. The results were illustrated with a microarray image. (E) The expression of eight circRNAs was further validated in paired metastatic or nonmetastatic tissues (mean ± SD; *n* = 60; Student's *t*‐test). (F) The circular structure of this circRNAs was analyzed and validated by Sanger sequencing. (G) PCR and an agarose gel electrophoresis assay were used to determine the expression level of back‐spliced or canonical forms of MMP2 in cDNA and gDNA of LM3 cell lines supplemented with or without RNase R. (H) qRT–PCR analysis examined the resistance of linear MMP2 or circular MMP2 to RNase R digestion (mean ± SD; *n* = 6; Student's *t*‐test). (I) The stability of circ_MMP2 or MMP2 mRNA in LM3 cells treated with actinomycin D (inhibitor of RNA synthesis) (mean ± SD; *n* = 6; two‐way ANOVA). *P* < 0.01 and *P* < 0.001 indicated data were statistically significant. n.s., no significance.

It has been reported that tumor cell‐derived exosomes can transfer differentially expressed genes to regulate cellular processes. In current study, we investigated whether LM3 and 97H cells can carry circRNAs. Through PKH67 staining, we identified the cup‐shaped structure and size of exosomes released by 97H and LM3 cells (Fig. 3A,B). In addition, the isolated particles were verified to be exosomes via detection of characteristic CD63 and CD81 (Fig. 3C). TAM was used to probe the exosomes derived from LM3 and U97H cells (Fig. S1A,B). Moreover, the IF staining of CD63 and CD81 was also used to detect the characteristic CD63 and CD81 (Fig. S1C). Confocal imaging of the delivery of Dio‐labeled exosomes to Dil‐labeled L02 and HepG2 cells was obtained (Fig. S1D). To confirm the function of exosomes delivered from 97H and LM3 cells, we also conducted *in vitro* and *in vivo* experiments in L02 and HepG2 cells under the condition of with or without 97H exosome. Through *in vitro* functional assay, we determined that cell migration, invasion, and EMT progress were promoted after L02 and HepG2 cells were treated with 97H exosome (Fig. S2A–C). Moreover, *in vivo* experiments demonstrated that tumor growth was accelerated by the treatment with 97H exosome (Fig. S2D–F). The lower positivity of E‐cadherin and higher positivity of N‐cadherin were measured in tumors derived from L02 and HepG2 cells treated without 97H exosome (Fig. S2G). Meanwhile, the metastatic nodules were found to be much less in control group than 97H exosome group (Fig. S2H). Next, microarray analysis was applied to identify circRNAs that were transferred by 97H‐ or LM3‐derived exosomes. The results indicated that there were eight circRNAs are significantly upregulated in exosomes (Fig. 3D). The expression of these eight circRNAs was further validated in paired metastatic or nonmetastatic tissues. Quantitative real‐time PCR (qRT–PCR) analysis showed that has_circ_0039411 (circ_MMP2) was markedly upregulated in metastatic tissues (Fig. 3E). The expression of circ_MMP2 was further measured in normal hepatic cell L02 and four HCC cells. As expected, circ_MMP2 was expressed at a higher level in HCC cells compared with L02 cell (Fig. S3A). Next, the circular structure of these circRNAs was analyzed and validated by Sanger sequencing (Fig. 3F). Through PCR and an agarose gel electrophoresis assay, we determined the expression level of back‐spliced or canonical forms of MMP2 in cDNA and gDNA of LM3 cell lines (Fig. 3G). Further qRT–PCR analysis revealed that circular MMP2, rather than linear MMP2, resisted RNase R digestion (Fig. 3H). Additionally, the stability of circ_MMP2 in LM3 cells treated with actinomycin D (inhibitor of RNA synthesis) was stronger than that of linear MMP2 (Fig. 3I). These findings suggested the delivery of exosome in 97H and LM3 cells.

### 3.4 Silencing of circ_MMP2 weakened metastatic ability of normal cell line and HepG2 cell line

**Figure 4:**
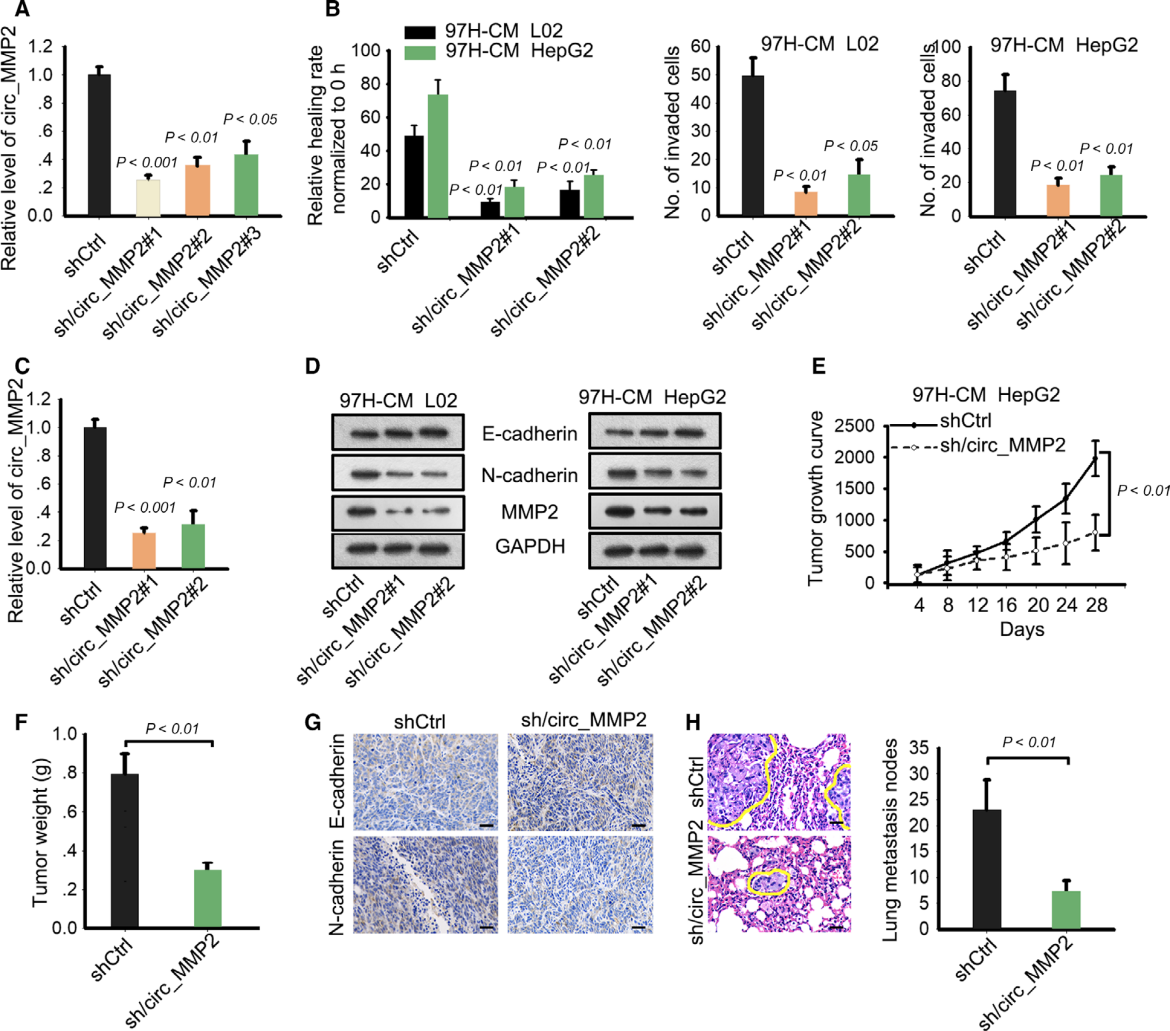
Silencing of circ_MMP2 weakened metastatic ability of normal cell line and HepG2 cell line. (A) Knockdown efficiency or circ_MMP2 in L02 and HepG2 cell lines treated with 97H‐CM (mean ± SD; *n* = 6; one‐way ANOVA). (B, C) Cell migration and invasion were observed in 97H‐cultured L02 and HepG2 cell lines after silencing of circ_MMP2 (mean ± SD; *n* = 60; Student's *t*‐test). (D) Protein level of E‐cadherin, N‐cadherin, and MMP2 was tested in cell lines transfected with circ_MMP2‐specific shRNAs. (E, F) Tumor growth was observed after knockdown of circ_MMP2 by calculating tumor volume (mean ± SD; *n* = 4; two‐way ANOVA) and tumor weight (mean ± SD; *n* = 4; Student's *t*‐test). (G) IHC staining revealed that the positivity of E‐cadherin and N‐cadherin in shCtrl or sh/circ_MMP2 group (scale bar = 50 μm; *n* = 4). (H) The effect of silencing of circ_MMP2 on lung metastasis nodes (scale bar = 50 μm; mean ± SD; *n* = 4; Student's *t*‐test). *P* < 0.05, *P* < 0.01, and *P* < 0.001 indicated data were statistically significant.

Based on the above findings, we confirmed that exosomes released by 97H and LM3 cell lines led to the malignant phenotype formation of normal liver cell line and contributed to metastasis of HCC cell lines. To demonstrate the role of circ_MMP2 in above biological processes, we performed loss‐of‐function assays. Knockdown efficiency of circ_MMP2 in two cultured cell lines was obtained 48 h after shRNA transfection (Fig. 4A,C). Subsequently, cell migration and invasion were observed in 97H‐cultured L02 and HepG2 cell lines after silencing of circ_MMP2. It was found that silencing of circ_MMP2 suppressed migration and invasion in both cell lines (Fig. 4B). Then, we observed that E‐cadherin level was increased, but N‐cadherin and MMP2 levels were decreased in cell lines transfected with circ_MMP2‐specific shRNAs (Fig. 4D). Additionally, *in vivo* experiments demonstrated that tumor growth was significantly inhibited by the knockdown of circ_MMP2 (Fig. 4E,F). IHC staining revealed that the positivity of E‐cadherin was increased, but the positivity of N‐cadherin was decreased in sh/circ_MMP2 group (Fig. 4G). Finally, we determined that lung metastasis nodes were reduced after silencing of circ_MMP2 (Fig. 4H). Thus, we confirmed circ_MMP2 delivered by exosomes released by 97H or LM3 cell line promoted the malignant phenotype formation of normal liver cell line and contributed to HCC metastasis.

### 3.5 circ_MMP2 regulated its host gene MMP2 via sequestering miR‐136‐5p

**Figure 5:**
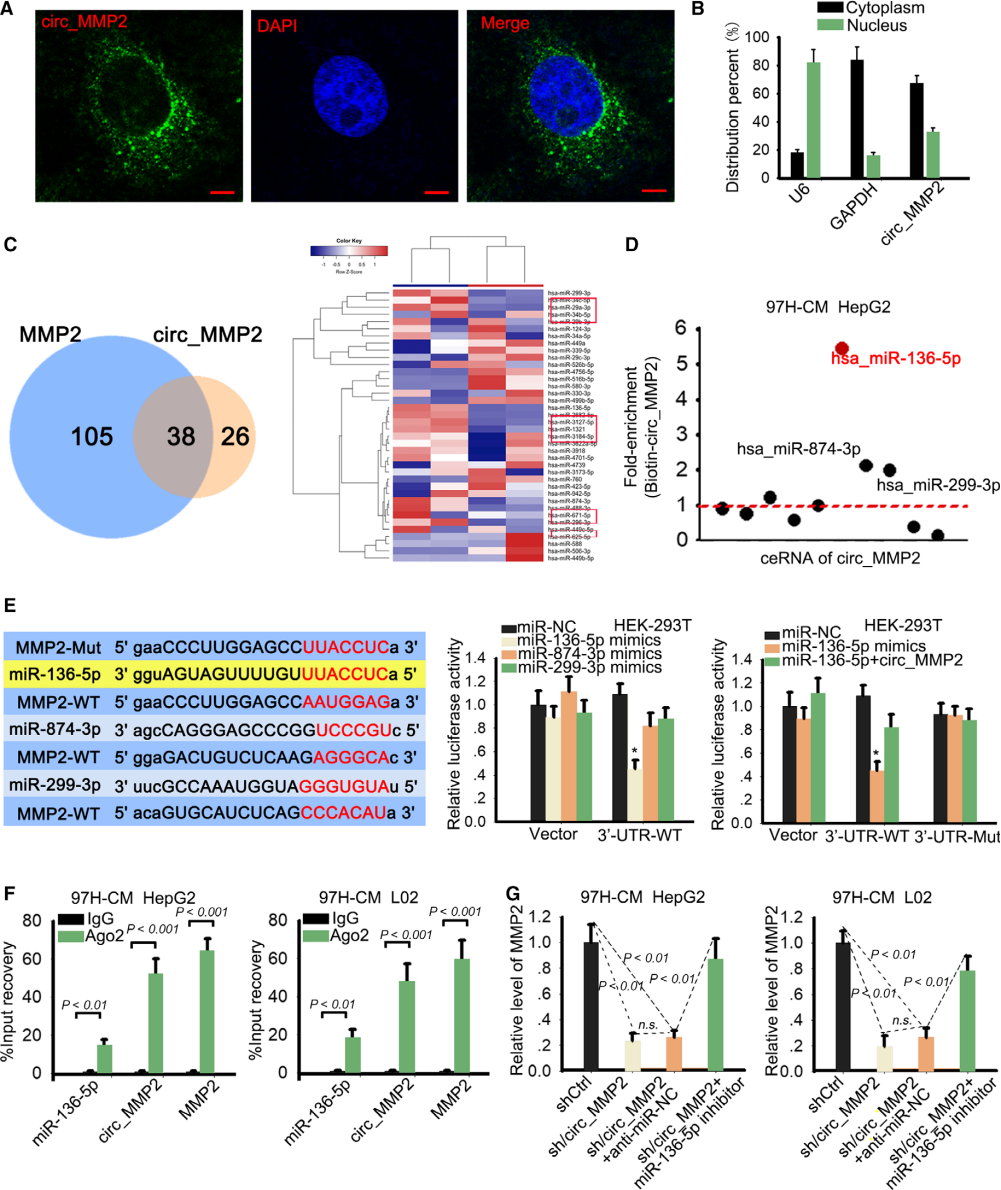
circ_MMP2 regulated its host gene MMP2 via sponging miR‐136‐5p. (A, B) Cytoplasmic localization of circ_MMP2 was determined by RNA FISH (scale bar = 10 μm; *n* = 5) and subcellular fractionation assay (*n* = 6). (C) 38 miRNAs predicted from StarBase were subjected to qRT–PCR analysis in original cell line or 97H‐CM‐cultured cell lines. (D) RNA pull‐down assay further demonstrated the enrichment of miR‐136‐5p, miR‐874‐3p, and miR‐299‐3p in the products pulled down by biotin‐circ_MMP2 probe (*n* = 6). (E) The binding sequences between these three miRNAs and MMP2‐WT or MMP2‐MUT were predicted and subjected to luciferase reporter assay (mean ± SD; *n* = 6; one‐way ANOVA). (F) The enrichment of circ_MMP2, miR‐136‐5p, and MMP2 in RISC was validated by Ago2‐RIP assay (mean ± SD; *n* = 6; Student's *t*‐test). (G) The expression of MMP2 in cell lines transfected with sh/circ_MMP2 or cotransfected with sh/circ_MMP2 and miR‐136‐5p inhibitor (mean ± SD; *n* = 6; one‐way ANOVA). *P* < 0.05, *P* < 0.01, and *P* < 0.001 indicated data were statistically significant. n.s., no significance.

To analyze the molecular mechanism of circ_MMP2 exerted in HCC metastasis, we detected its cellular localization. Based on RNA FISH and subcellular fractionation assay, we identified that circ_MMP2 was predominantly located in cytoplasm of HCC cell lines (Fig. 5A,B), suggesting the post‐transcriptional regulation of circ_MMP2. circRNAs can exert oncogenic functions via regulating their host gene in ceRNA models (Li *et al.*, 2018a; Qiu *et al.*, 2018). MMP2 is acknowledged as an oncogene that is correlated with metastasis. According to above data, we determined that circ_MMP2 positively regulated MMP2. Therefore, we investigated whether circ_MMP2 can exert ceRNA function to regulate MMP2. According to the search results of StarBase database (http://starbase.sysu.edu.cn/), there are 38 miRNAs that have complementary base pairing with both circ_MMP2 and MMP2 (Fig. 5C, left). Thirty‐eight miRNAs were subjected to qRT–PCR analysis in original cell line or 97H‐CM‐cultured cell lines, and the results showed that ten miRNAs were significantly downregulated in cell lines cultured in 97H‐CM (Fig. 5C, right). Through luciferase reporter assay, we excluded that circ_MMP2 could directly regulate MMP2 expression by transcriptional regulation (Fig. S3B). Pull‐down assay further demonstrated that miR‐136‐5p, miR‐874‐3p, and miR‐299‐3p presented the highest enrichment in the products pulled down by biotin‐circ_MMP2 probe (Fig. 5D). The binding sequences between these three miRNAs and MMP2‐WT or MMP2‐MUT were predicted and subjected to luciferase reporter assay (Fig. 5E). It was found that the luciferase activity of wild‐type MMP2 3′UTR (MMP2‐WT) was significantly decreased by the overexpression of miR‐136‐5p mimics. We also performed qRT–PCR to quantify the exact copy numbers of circ_MMP2 and miR‐136‐5p in HCC cells and L02 cell. The results are shown in Fig. S3C. High copy number was found in 97H and LM3 cells, and the copy number of circ_MMP2 was more than miR‐136‐5p in 97H and LM3 cells. Importantly, the decreased luciferase activity was recovered by the introduction of circ_MMP2 expression vector. There was no obvious change in the luciferase activity of mutant type circ_MMP2 (MMP2‐MUT). Ago2‐RIP assay further validated the enrichment of circ_MMP2, miR‐136‐5p, and MMP2 in RISC (Fig. 5F), indicating the ceRNA network was formed. Finally, we examined the expression of MMP2 in response to the expression change in circ_MMP2 or miR‐136‐5p. As displayed in Fig. 5G, the expression of MMP2 was impaired by the knockdown of cric_MMP2, while this tendency was reversed partially by the cotransfection of miR‐136‐5p inhibitor.

### 3.6 Clinical significance of circ_MMP2/miR‐136‐5p/MMP2 axis in HCC

**Figure 6:**
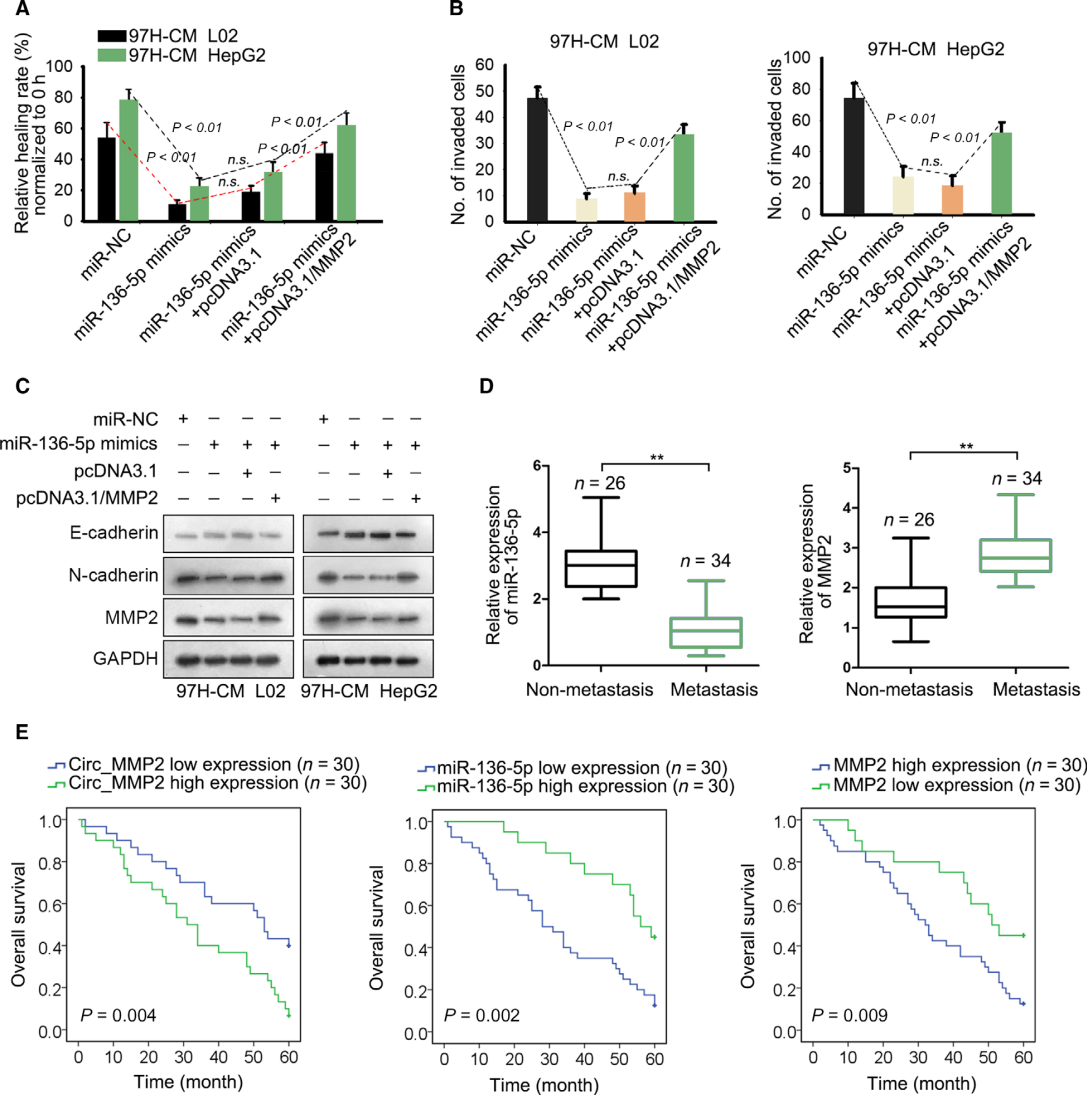
MMP2 and miR‐136‐5p are crucial participants in HCC metastasis. (A, B) Cell migration and invasion were examined in 97H‐CM‐cultured L02 and HepG2 cell lines after transfecting with miR‐136‐5p mimics or cotransfected with pcDNA3.1/MMP2 (mean ± SD; *n* = 6; one‐way ANOVA). (C) The level of E‐cadherin, N‐cadherin, and MMP2 was examined in indicated cell lines. (D) Expression level of miR‐136‐5p or MMP2 in HCC tissues with or without metastasis (mean ± SD; *n* = 60; Student's *t*‐test). (E) Overall survival rate of HCC patients with high or low level of circ_MMP2, miR‐136‐5p, or MMP2 (Kaplan–Meier method followed by log‐rank test). *P* < 0.01 indicated data were statistically significant. n.s., no significance. **means *P* < 0.01.

Functional rescue assays were conducted to demonstrate the role of miR‐136‐5p and MMP2 in HCC metastasis. Intriguingly, cell migration and invasion were significantly suppressed after transfection of miR‐136‐5p mimics. However, decreased cell migration and invasion were recovered after cotransfection with MMP2 expression vector (Fig. 6A,B). Additionally, increased E‐cadherin level and decreased N‐cadherin level were measured in cell lines transfected with miR‐136‐5p mimics (Fig. 6C), whereas this tendency was reversed by the introduction of MMP2. To assess the clinical significance of circ_MMP2, miR‐136‐5p, or MMP2 in HCC, we measured the expression level of them in different HCC samples. Intriguingly, relative low level of miR‐136‐5p and the high level of MMP2 were observed in HCC tissues with metastasis (Fig. 6D). Next, all HCC tissue specimens were classified into different groups in accordance with the median value of circ_MMP2, miR‐136‐5p, or MMP2 expression. After Kaplan–Meier analysis, we determined that patients with high level of circ_MMP2 or MMP2 had a lower survival rate than those with low level (Fig. 6E). Additionally, the overall survival of HCC patients with low miR‐136‐5p level was worse than those with high level. Therefore, we confirmed that circ_MMP2 transmitted by 97H‐derived exosomes promoted HCC metastasis by sponging miR‐136‐5p to upregulate MMP2.

## 4 Discussion

Hepatocellular carcinoma is a common malignancy that has become a challenge to public health. In this study, we examined the malignant degree of different HCC cell lines and measured the metastatic ability of both normal liver cell line and HCC cell lines. As expected, HCC cell lines had stronger metastasis than normal cell line. Since 97H is the cell line with highest metastasis, we used the culture medium of 97H cell lines to incubate both normal cell line and HCC cell line. *In vitro* and *in vivo* experiments supported that cell lines cultured with 97H‐CM had high metastasis. More importantly, treatment with 97H‐CM led to the malignant formation of normal liver cell line.

Tumor microenvironment mediated by intercellular communications is essential for tumor progression and metastasis. As an element of dynamic system, exosome can deliver the biological molecules into tumor cells, thus promoting tumorigenesis or tumor progression (Hwang *et al.*, 2019; Uotani *et al.*, 2017). Herein, investigating the interaction between tumor progression and exosome‐mediated stroma is necessary for cancer research. Based on our experimental data, we investigated whether 97H‐CM containing exosome that can deliver biological molecules to affect HCC metastasis. In the current study, we determined that 97H and LM3 cell lines carried circ_MMP2 and transferred it into L02 and HepG3 cell lines. High level of circRNAs is correlated with high metastasis and fast tumor progression (Chen *et al.*, 2017, 2018; Hsiao *et al.*, 2017b; Shen *et al.*, 2019). Here, we conducted loss‐of‐function assays and uncovered that silencing of circ_MMP2 efficiently suppressed HCC metastasis and reversed the malignancy of normal L02 cell line.

Mechanistically, circRNAs can regulate its host gene expression at post‐transcriptional level. Our research findings suggested that MMP2 is positively regulated by circ_MMP2. MMP2, a metastasis‐related protein, promotes tumor metastasis (Chen *et al.*, 2013; Incorvaia *et al.*, 2007; Rahme and Israel, 2015; Zhu *et al.*, 2017). Therefore, we explored whether circ_MMP2 positively regulated MMP2 via sequestering miRNAs. After bioinformatic analysis and mechanism experiments, we identified that miR‐136‐5p could bind to both circ_MMP2 and MMP2. Hereto, we determined that circ_MMP2 acted as a ceRNA to regulate its host gene MMP2 by sequestering miR‐136‐5p. Next, rescue assays demonstrated that miR‐136‐5p exerted tumor‐suppressive role in HCC metastasis. Importantly, the suppressive function of miR‐136‐5p was recovered by the upregulation of MMP2.

**Figure 7:**
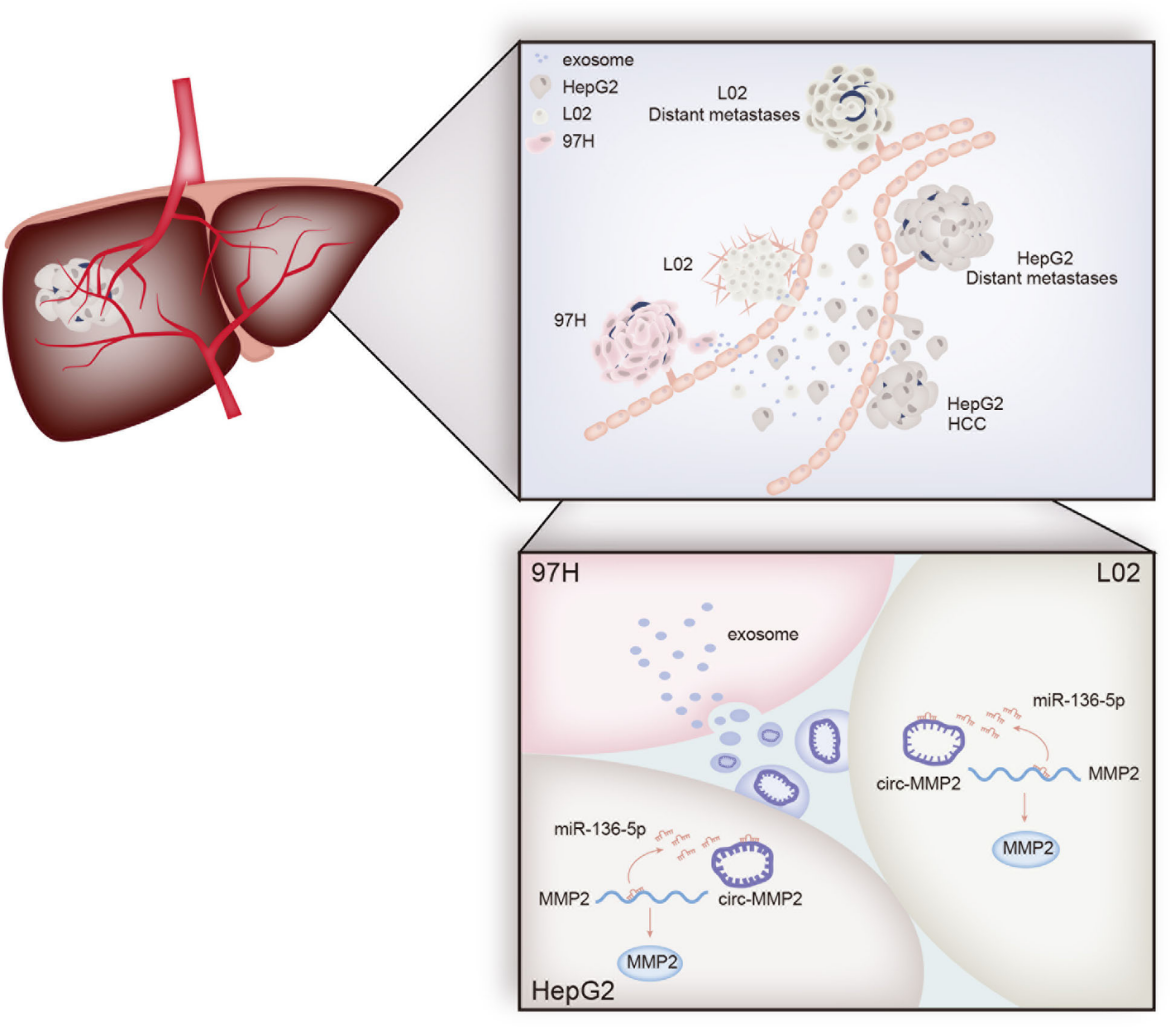
circ_MMP2 was delivered from 97H‐derived exosome into L02 and HepG2 cells and promoted tumor metastasis and EMT process.

Increasing studies reported that circRNAs and their downstream miRNAs or mRNAs are clinically significant (Ding *et al.*, 2018; Xia *et al.*, 2016; Yang *et al.*, 2018; Zhang *et al.*, 2017). In our present study, we examined high expression of circ_MMP2 and MMP2 in metastatic samples. More importantly, upregulation of circ_MMP2 and MMP2 was associated with low overall survival rate of HCC patients. Therefore, we determined the clinical significance of circ_MMP2 and MMP2 in HCC patients. In conclusion, this study identified a novel ceRNA pathway in 97H‐derived exosome (Fig. 7). Our research findings may contribute to explore biomarkers in HCC treatment.

## 5 Conclusion

High level of circ_MMP2 and its linear mRNA MMP2 was identified in metastatic HCC samples. Upregulation of circ_MMP2 and MMP2 indicated poor overall survival rate of HCC patients. circ_MMP2 was delivered by 97H‐ or LM3‐secreted exosomes into L02 and HepG2 cells and promoted metastasis in HCC by sponging miR‐136‐5p to enhance MMP2 expression. Our findings suggested that circ_MMP2 may be a novel biomarker in HCC treatment.

## Conflict of interest

The authors declare no conflict of interest.

## Author contributions

DL designed the experiments. DL, HK, and MG performed the experiments. LJ and FZ collected the clinical samples and analyzed the data. DL and DC prepared figures and drafted the manuscript. ML supervised laboratorial processes. LX provided the revision proposal and research fund. All authors read and approved the final manuscript.

## Ethic statement

All procedures of this study were approved by the Ethical Committee of the First Hospital of Lanzhou University. Before this study, every patient enrolled in this study had given the written informed consent. Animal experiments conducted in this study had been approved by the First Hospital of Lanzhou University.

## Supporting information


**Fig. S1.** Identification of exosomes. (A, B) Electron microscope (scale bar = 200 nm; n = 3) and Nanosight particle tracking analysis were applied to detect the exosomes derived from LM3 and 97H. (C) Confocal imaging of the delivery of Dio‐labeled exosomes to Dil‐labeled L02 and HepG2 cells (scale bar = 50 μm; n = 5). (D) Immunofluorescence staining of characteristic CD63 and CD81 (scale bar = 20 μm; n = 5).

**Fig. S2.** 97H‐secreted exosomes promoted the growth and metastasis in L02 and HepG2 cells. (A, B) Migration and invasion of L02 and HepG2 cells treated with or without 97H‐exosome were separately assessed (mean ± SD; n = 6; Student's t‐test). (C) The levels of E‐cadherin and N‐cadherin in 97H‐exosome or control group. (D) Animal study revealed that tumor growth in 97H exosome group was faster than control group. (E, F) Tumor volume (mean ± SD; n = 4; two‐way ANOVA) and tumor weight (mean ± SD; n = 4; Student's t‐test) were measured in two different groups. (G) Positivity of E‐cadherin or N‐cadherin in 97H‐CM group or control group was assessed by IHC staining (scale bar = 50 μm; n = 4). (H) Lung metastasis nodes 97H‐CM group or control group was measured and counted (scale bar = 50 μm; mean ± SD; n = 4; Student's t‐test).* p *< 0.05, *p *< 0.01 indicated data were statistically significant.

**Fig. S3.** circ_MMP2 expression and its regulatory effect on MMP2 transcription. (A) Northern blot analysis of circ_MMP2 expression in L02 cell and four HCC cells. (B) Luciferase activity analysis was used to assess the effect of circ_MMP2 on the transcription activity of MMP2 (mean ± SD; n = 6; one‐way ANOVA). (C) Copy number of circ_MMP2 and miR‐136‐5p was evaluated in L02, HepG2, 97H and LM3 cells by qRT–PCR (mean ± SD; n = 6; Student's t‐test). *p *< 0.05 indicated data were statistically significant. n.s.: no significance.

**Table S1.** Primers for qRT–PCR analyses.

## Data Availability

Research data are available.
