# Supplementary figures and images for: Exosome‐transmitted circ_MMP2 promotes hepatocellular carcinoma metastasis by upregulating MMP2
Source: Mol Oncol. 2020 May 6;14(6):1365–80. doi: 10.1002/1878-0261.12637 (PMC7266270; doi:10.1002/1878-0261.12637)

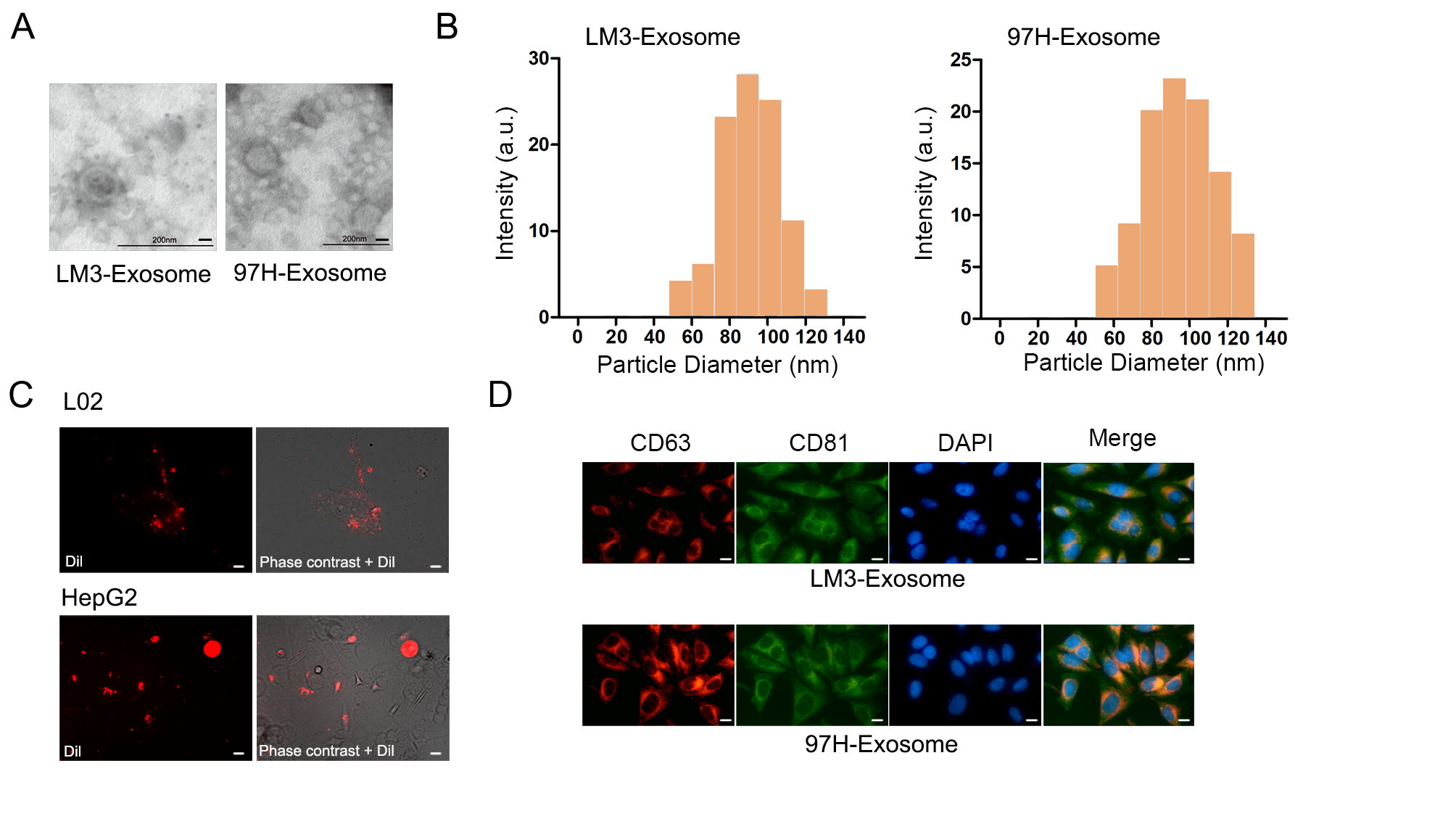

Supplement: Supplementary file 1 — Fig. S1. Identification of exosomes. (A, B) Electron microscope (scale bar = 200 nm; n = 3) and Nanosight particle tracking analysis were applied to detect the exosomes derived from LM3 and 97H. (C) Confocal imaging of the delivery of Dio‐labeled exosomes to Dil‐labeled L02 and HepG2 cells (scale bar = 50 μm; n = 5). (D) Immunofluorescence staining of characteristic CD63 and CD81 (scale bar = 20 μm; n = 5). [file MOL2-14-1365-s001.tif]

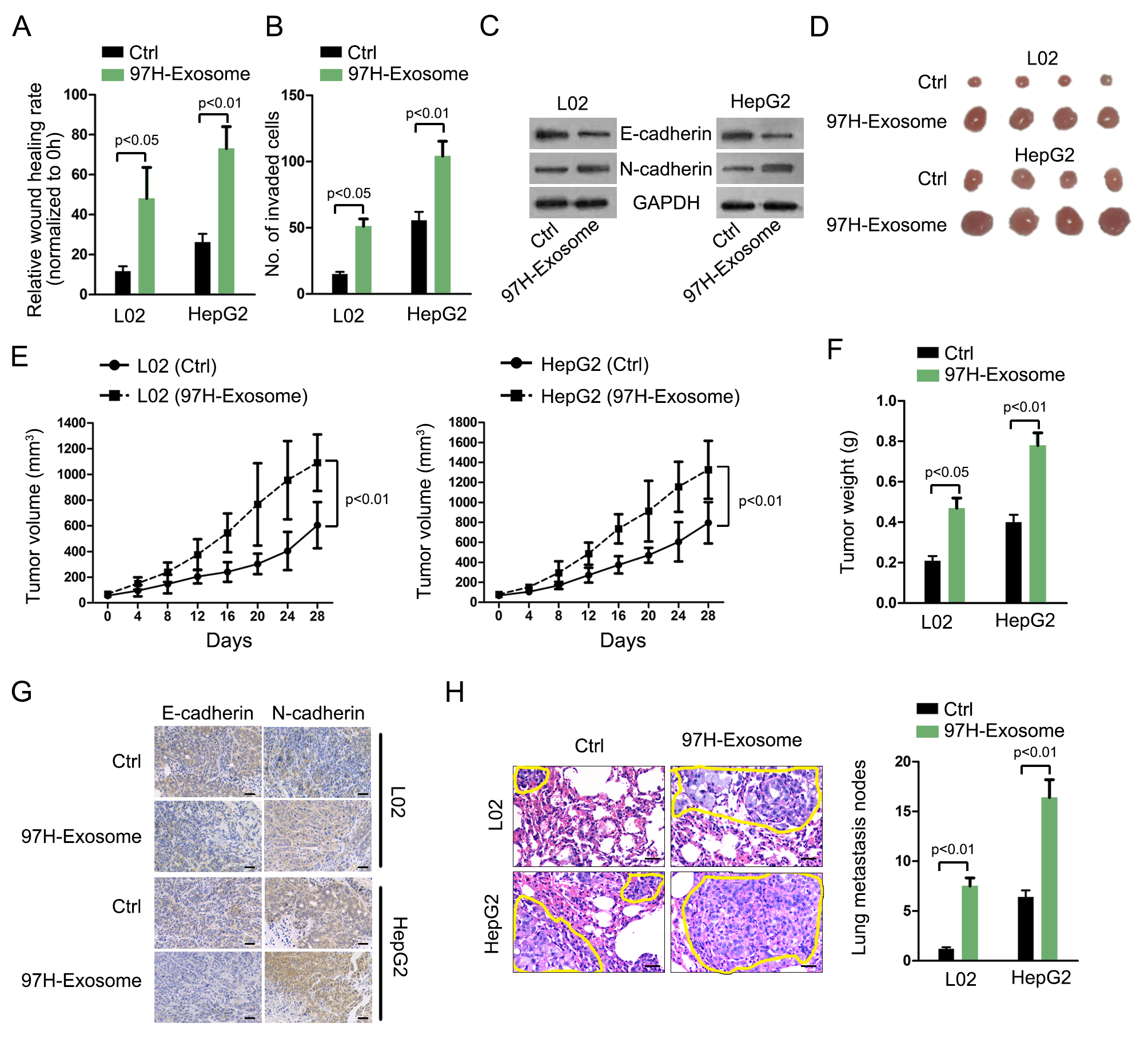

Supplement: Supplementary file 2 — Fig. S2. 97H‐secreted exosomes promoted the growth and metastasis in L02 and HepG2 cells. (A, B) Migration and invasion of L02 and HepG2 cells treated with or without 97H‐exosome were separately assessed (mean ± SD; n = 6; Student's t‐test). (C) The levels of E‐cadherin and N‐cadherin in 97H‐exosome or control group. (D) Animal study revealed that tumor growth in 97H exosome group was faster than control group. (E, F) Tumor volume (mean ± SD; n = 4; two‐way ANOVA) and tumor weight (mean ± SD; n = 4; Student's t‐test) were measured in two different groups. (G) Positivity of E‐cadherin or N‐cadherin in 97H‐CM group or control group was assessed by IHC staining (scale bar = 50 μm; n = 4). (H) Lung metastasis nodes 97H‐CM group or control group was measured and counted (scale bar = 50 μm; mean ± SD; n = 4; Student's t‐test). p < 0.05, p < 0.01 indicated data were statistically significant. [file MOL2-14-1365-s002.tif]

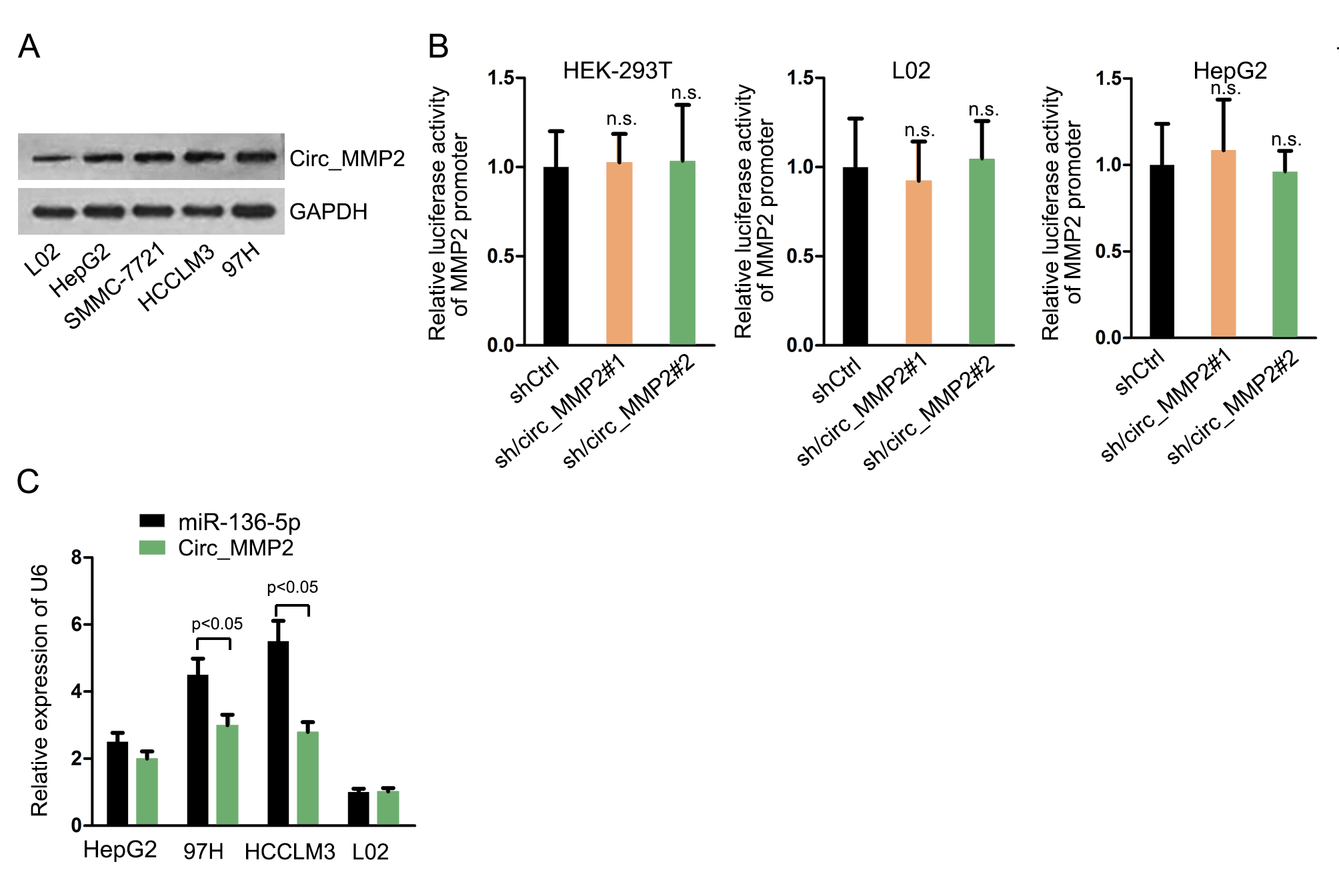

Supplement: Supplementary file 3 — Fig. S3. circ_MMP2 expression and its regulatory effect on MMP2 transcription. (A) Northern blot analysis of circ_MMP2 expression in L02 cell and four HCC cells. (B) Luciferase activity analysis was used to assess the effect of circ_MMP2 on the transcription activity of MMP2 (mean ± SD; n = 6; one‐way ANOVA). (C) Copy number of circ_MMP2 and miR‐136‐5p was evaluated in L02, HepG2, 97H and LM3 cells by qRT–PCR (mean ± SD; n = 6; Student's t‐test). p < 0.05 indicated data were statistically significant. n.s.: no significance. [file MOL2-14-1365-s003.tif]
